# Optimizing the alignment of thermoresponsive poly(N-isopropyl acrylamide) electrospun nanofibers for tissue engineering applications: A factorial design of experiments approach

**DOI:** 10.1371/journal.pone.0219254

**Published:** 2019-07-05

**Authors:** Rachel E. Young, Jodi Graf, Isabella Miserocchi, Ryan M. Van Horn, Melissa B. Gordon, Christopher R. Anderson, Lauren S. Sefcik

**Affiliations:** Department of Chemical and Biomolecular Engineering, Lafayette College, Easton, Pennsylvania, United States of America; University of Fukui, JAPAN

## Abstract

Thermoresponsive polymers, such as poly(N-isopropyl acrylamide) (PNIPAM), have been identified and used as cell culture substrates, taking advantage of the polymer’s lower critical solution temperature (LCST) to mechanically harvest cells. This technology bypasses the use of biochemical enzymes that cleave important cell-cell and cell-matrix interactions. In this study, the process of electrospinning is used to fabricate and characterize aligned PNIPAM nanofiber scaffolds that are biocompatible and thermoresponsive. Nanofiber scaffolds produced by electrospinning possess a 3D architecture that mimics native extracellular matrix, providing physical and chemical cues to drive cell function and phenotype. We present a factorial design of experiments (DOE) approach to systematically determine the effects of different electrospinning process parameters on PNIPAM nanofiber diameter and alignment. Results show that high molecular weight PNIPAM can be successfully electrospun into both random and uniaxially aligned nanofiber mats with similar fiber diameters by simply altering the speed of the rotating mandrel collector from 10,000 to 33,000 RPM. PNIPAM nanofibers were crosslinked with OpePOSS, which was verified using FTIR. The mechanical properties of the scaffolds were characterized using dynamic mechanical analysis, revealing an order of magnitude difference in storage modulus (MPa) between cured and uncured samples. In summary, cross-linked PNIPAM nanofiber scaffolds were determined to be stable in aqueous culture, biocompatible, and thermoresponsive, enabling their use in diverse cell culture applications.

## Introduction

For decades now, tissue engineers have developed myriad ways to regenerate or replace damaged tissues and organs with tissue-engineered constructs. In the case of cell-based constructs, expanding cells *in vitro* for eventual *in vivo* transplantation is hindered by using biochemical enzymes to release cells from tissue culture plastic. These enzymes (i.e. tryspin, EDTA) degrade cell-cell and cell-matrix interactions, which are critical mediators in cellular function and phenotype. To circumvent the use of enzymes, thermoresponsive polymers have been developed and extensively used as cell culture substrates due to their temperature dependent physico-chemical properties. One thermoresponsive cell culture substrate, Nunc UpCell, is commercially available and features covalently grafted poly(N-isopropylacrylamide) (PNIPAM). Temperature-responsive PNIPAM exhibits a reversible phase change at its lower critical solution temperature (LCST) of 32°C. Above 32°C, PNIPAM is hydrophobic, supporting protein adsorption and cell adhesion and proliferation. Upon a temperature switch below 32°C, PNIPAM is hydrophilic and the rapid hydration of the polymer network results in cellular detachment [1]. Use of thermoresponsive polymers for cell culture substrates, such as UpCell, allows for the noninvasive collection of a cell monolayer with preservation of the extracellular matrix (ECM) [2,3] and important cell-cell junctions; therefore increasing the probability of successful implantation and host integration [4].

The ECM has important nanofibrous architecture that governs cell adhesion, proliferation, migration, and differentiation [5–7]. Because the ECM also anchors and supports cells physically, it is desirable to create tissue-engineered scaffolds that mimic native ECM. Various processing techniques have been introduced to fabricate nanofiber scaffolds, such as phase separation, self-assembly, and electrospinning, the latter attracting significant attention because of its ease-of-use and cost-effectiveness. The process of electrospinning creates 3D nanofibrous, porous mats with a high surface area to volume ratio, allowing for increased cell binding sites and transport of oxygen or growth factors through the scaffold. This high surface area to volume ratio has also proven beneficial in the specific case of PNIPAM; it has been demonstrated that increasing the surface area to volume ratio of PNIPAM substrates resulted in faster cellular release times compared to 2D PNIPAM constructs [8]. Several studies have reported on successful electrospinning of PNIPAM, utilizing different molecular weights, solvents, and electrospinning parameters [2,9–12]. Cicotte et al. found that electrospun scaffolds of high molecular weight PNIPAM (300 kDa) were thermoresponsive, biocompatible, and stable in aqueous culture, but scaffolds of LMW PNIPAM (40 kDa) dissolved instantly upon aqueous immersion [10]. Allen et al. successfully generated HMW PNIPAM/PCL co-fibers that were biocompatible, yet reported impaired cell viability on 100% PNIPAM scaffolds [12]. Although neither of these studies cross-linked the PNIPAM mats, it is known that linear PNIPAM is soluble in water [9,13]. Electrospun PNIPAM nanofibers would therefore lose their desirable 3D architecture upon introduction to aqueous culture. To circumvent this issue, Wang et al. developed a methodology to cross-link PNIPAM nanofibers using Octaglycidyl polyhedral oligomeric silsesquioxane (OpePOSS) as a crosslinker with a strong base catalyst (2-ethyl-4-methylimidazole, EMI), followed by heating. Their optimized approach produced highly stable and robust thermoresponsive PNIPAM nanofibers [14].

Apart from the PNIPAM/PCL co-fibers reported by Allen et al. in 2017, electrospun PNIPAM nanofibers have been collected as nonwoven mats on grounded collector plates. The resulting random fiber orientation, albeit 3D, biocompatible, and thermoresponsive, does not mimic the anisotropic architecture of native ECM found in tissues such as nerve, heart, and muscle. However, by placing a rotating mandrel in the electric field gradient during the electrospinning process, nanofibers can be aligned in the direction of rotation, thereby providing the biophysical cues that drive cellular alignment, guide cell migration and extension, and subsequently influence cell phenotype that more closely mimics native tissues [15–17]. Thus, the present study optimizes the electrospinning process to create thermoresponsive, aligned PNIPAM nanofibers capable of supporting cell adhesion and proliferation for use in tissue engineering applications where uniaxial alignment is ideal, such as vascular, bone, or neural grafts.

In this study, we employ factorial design of experiments (DOE) to systematically determine the effects of different electrospinning process parameters on PNIPAM nanofiber diameter and alignment. Few studies have utilized a DOE for the optimization of electrospun nanofiber diameter and alignment [18–21]. Here, we present the range of parameters tested and aim to highlight the robustness of a statistical DOE approach in identifying an optimal parameter set for the electrospinning of PNIPAM. We demonstrate that HMW PNIPAM can be electrospun into random or uniaxially aligned nanofiber mats by simply altering speed of the rotating mandrel collector. Importantly, fiber diameter was the same for random vs. aligned fibers, allowing for an accurate comparison in future studies that examine the effects of alignment on cell function and phenotype. The optimized cross-linked nanofibers were stable in aqueous culture, biocompatible, and thermoresponsive.

## Materials and methods

### Materials

High molecular weight (HMW) poly(N-isopropyl acrylamide) (PNIPAM, MW = 300,000 Da) was purchased from Scientific Polymers (Ontario, NY). Octaglycidyl polyhedral oligomeric silsequioxane (OpePOSS) was purchased from Hybrid Plastics Inc. (Hattiesburg, MS). 2-ethyl-4-methylimidazole (EMI), tetrahydrofuran (THF), and dimethylformamide (DMF) were purchased from Sigma Aldrich (St. Louis, MO). PNIPAM, OpePOSS, and EMI were dissolved by vortexing in 1:1 THF: DMF (by volume) at room temperature at a ratio 100:20:0.3, respectively. The final PNIPAM concentration was 12% by mass. The following materials were used for cell culture: Dulbecco’s Modified Eagle Medium (DMEM) from Gibco (11885–084), 0.25% trypsin/2.21mM EDTA 1x from Corning (25-053-CI), penicillin streptomycin solution 100x from Corning (30-001-CI), and Qualified, US Origin fetal bovine serum from Gibco (26-140-079). Nunc 48-well multidishes with UpCell surface were purchased from Thermo Scientific.

### Nanofiber fabrication and characterization

#### Fiber morphology optimization

A vertical electrospinning set-up was utilized to fabricate PNIPAM nanofibers. Voltage was supplied by a DC power supply (Omega High Voltage Research, FL), and flow rate was controlled using a syringe pump (New Era Pump Systems, NY). All samples were collected on a grounded collector plate, wrapped in aluminum foil, and vacuum desiccated overnight before further use. A fractional factorial design of experiment (DOE) with three factors and two levels was constructed to identify electrospinning process parameters influencing PNIPAM fiber diameter. The factors and levels examined were: collecting distance (10 and 20cm), applied voltage (10 and 25 kV), and syringe pump flow rate (0.1 and 1.0 mL/hr). The PNIPAM solution was loaded into a 5 mL glass syringe, fitted with a 22-gauge (0.41mm ID) needle.

#### Imaging and analysis

The morphologies of nonwoven and aligned PNIPAM nanofiber scaffolds were analyzed using a scanning electron microscope (SEM; Phenom ProX microscope) at an accelerated voltage of 5 kV). Prior to imaging, samples were coated with 250 Angstroms of gold via a Denton Desk V Sputter Coater. Fiber diameters were quantified using the SEM micrographs and Image J software (NIH). For each sample, 5 random field images were taken and 20 fibers were measured in each image. Fiber alignment was quantified as a coherency value using the Orientation J plug-in for the dominant direction in Image J [22].

#### Aligned fiber optimization

A rotating mandrel design was utilized in conjunction with the vertical electrospinning set-up described above to fabricate a PNIPAM scaffold with aligned nanofibers. An aluminum mandrel was placed between the needle tip and grounded collector plate to interrupt the electric field and collect the fibers. Two different mandrel set-ups were used: an IKA RW 16 basic overhead stirrer with speeds ranging between 50 and 1545 RPM and a Dremel 300 with variable speed range of 10,000 to 33,000 RPM. Two factor, two-level DOE was designed to optimize nanofiber alignment and diameter. In the first study, mandrel diameter was varied from 0.75 inches (19mm) to 1.25 inches (32mm) and mandrel speed from 675 RPM to 1356 RPM. The collecting distance, applied voltage, and syringe pump flow rate were held constant at 13 cm, 15 kV, and 0.1 mL/hr, respectively. In the second study, mandrel diameter was varied from 0.156 inches (4mm) to 0.195 inches (5mm) and mandrel speed from 10,000 RPM to 33,000 RPM. Collecting distance, applied voltage, and syringe pump flow rate were held constant at 11 cm, 20 kV, and 0.1 mL/hr, respectively. The PNIPAM solution was loaded into a 5 mL glass syringe, fitted with a 22-gauge needle. To remove nanofibers from the mandrel, the scaffold was carefully cut using a razor blade along the length of the mandrel, unwrapped, placed on aluminum foil and vacuum desiccated overnight.

#### Crosslinking reaction

After dessication overnight, electrospun scaffolds of PNIPAM:OpePOSS:EMI were chemically crosslinked to improve the stability of the scaffold in aqueous solutions, based on the methods described by Wang et al. [14]. All samples were cured in an oven at 120°C for 4 hours. After curing, samples were stored in a dessicator until needed.

#### Fourier-transform infrared spectroscopy

To verify inclusion of OpePOSS and reaction of epoxide functionalities, attenuated total reflectance (ATR)-FTIR was performed on dried nanofibers using a ThermoScientific Nicolet iS50 spectrometer with a single-bounce diamond ATR crystal. The spectra were collected over a range of wavenumbers (600 to 4000 cm^-1^) with 16 scans and a resolution of 8 (0.964 cm^-1^). To verify the crosslinking reaction extended beyond the material surface, transmission-mode FTIR experiments were conducted using the Nicolet iS50 equipped with a DTGS KBr detector. Spectra were collected using 16 scans with a resolution of 8 and analyzed with Omnic software (Nicolet).

#### Dynamic mechanical analysis

DMA measurements were performed on a TA Instruments Q800 dynamic mechanical analyzer. Samples were measured using a strain amplitude of 0.10% at 1 Hz with a preload force of 0.1 N in oscillatory film tension mode at room temperature in the unaligned direction. Reported values contain averaged data of storage modulus values measured in triplicate from three independently prepared samples. Error bars represent standard error of the mean.

#### Aqueous stability and phase transition

The stability of the scaffold in aqueous solutions was quantified by measuring mass loss over time. The cured samples were first massed and then immersed in deionized water at 37°C for 24 hours and 7 days. The samples were removed from the water and vacuum desiccated for 48 hours to ensure complete water removal. The samples were massed again after drying. These experiments were conducted in triplicate. To qualitatively assess scaffold stability and physically observe the phase transition due to a temperature shift, the cured samples were immersed in deionized water and incubated overnight at 37°C and 5% CO_2_. The samples were removed from the incubator and allowed to cool to room temperature (~22°C) (below their LCST) to facilitate the hydrophobic-hydrophilic phase transition. This phase transition of the immersed samples was qualitatively assessed by observing the phase change of the scaffold from relatively opaque above the LCST to relatively transparent below the LCST.

### Cell studies

#### Thermoresponsive cell release

Cell studies were performed using murine L-929 fibroblasts (ATCC) cultured in DMEM, supplemented with 10% fetal bovine serum and 1% penicillin/streptomycin. L-929 fibroblasts were passaged using trypsin/EDTA. Nanofiber scaffolds were cut into 1.0 cm-diameter circles using a biopsy punch and fixed to the bottom of a 48-well plate with vacuum grease to prevent them from floating. TCP and PNIPAM UpCell were used as controls. All wells (n = 3 per group) were incubated overnight at 37°C and 5% CO_2_ in full serum media for preconditioning prior to cell-seeding [23]. Cells were seeded at a density of 30,000 cells/cm^2^. After an initial 16 hour attachment period (i.e. overnight), the attached cells were washed with room temperature media (~22°C) to facilitate temperature-stimulated detachment for 5 minutes. After the 5 minute detachment period, the supernatant was transferred to an empty well of the culture plate. The number of viable cells in the supernatant was determined using a CellTiter 96 AQ_ueous_ One Solution Cell Proliferation Assay (Promega, WI), according to the manufacturer’s directions. Warmed culture medium (37°C) was added to the original cell-seeded wells, along with MTS reagent, to measure the number of viable cells still attached to the substrate. After the 2-hour MTS incubation period, 100ul of solution from each experimental well was measured using a BioTek Synergy HT Microplate Reader, and absorbance at 490nm was converted to cell number using a standard curve. The percentage of thermoresponsive cell release was calculated as the number of cells released divided by the total cell number (cells released plus cells remaining).

#### Cell proliferation assay

To measure cell growth over the course of 48 hours, TCP, UpCell, and nanofiber scaffolds were preconditioned as described above and seeded with L-929 fibroblasts at a density of 25,000 cells/cm^2^ in triplicate. The number of viable cells was determined at each time-point using the MTS assay. After 4, 24, and 48 hours in culture, sample media was refreshed and MTS reagent was added. After the 2-hour incubation period at 37°C, 100 μL of supernatant was transferred to a 96 well plate and absorbance was measured at 490nm using the BioTek plate reader.

### Statistical analyses

Minitab 18 Statistical Software was used for all DOE design and statistical data analysis. 2-sample t-tests were used to determine significance between two experimental groups, while single-factor analysis of variance (ANOVA) coupled with Tukey’s post hoc test was employed to assess statistical difference between three or more sample groups. General Linear Model Regression Analysis was used to determine significance in the slopes of the linearized cell growth model. Statistical significance was asserted at p-value < 0.05.

## Results and discussion

### Nanofiber fabrication

**Fig 1:**
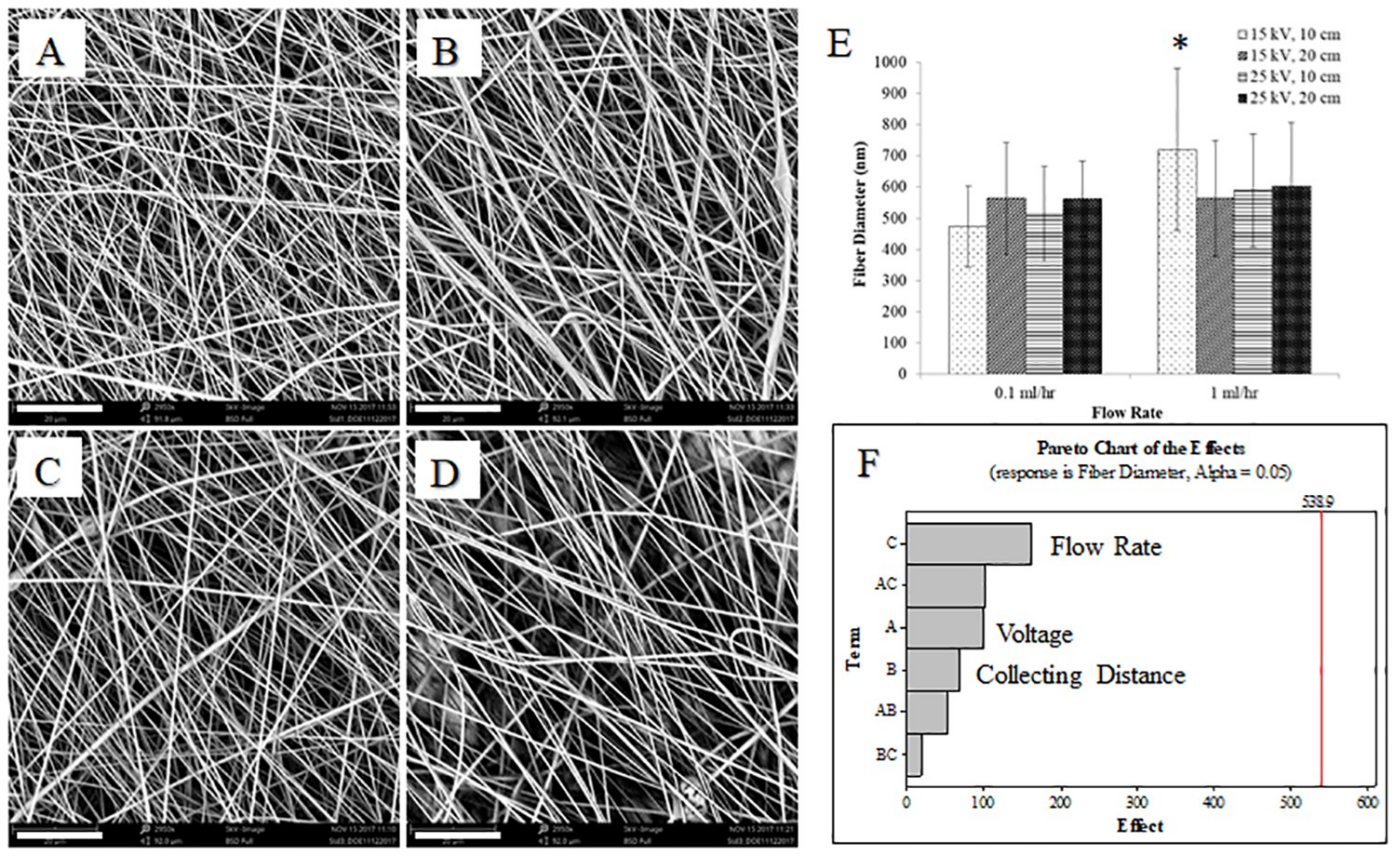
Non-woven PNIPAM nanofiber diameter as a function of collecting distance, flow rate, and voltage. The images in panels A-D are samples at the 0.1 ml/hour flow rate. (A) 10 cm collecting distance, 15 kV applied voltage. (B) 10 cm, 25 kV. (C) 20 cm, 15 kV. (D) 20 cm, 25 kV. Scale bar (lower left of each image) is 20 μm. Electrospinning parameters held constant: 12% (w/w) PNIPAM:OpePOSS:EMI (100:20:0.3) in a solution of THF:DMF (1:1), 22 ga. needle. (E) Quantitative effects of process parameters on fiber diameter. * p<0.05 relative to other samples at 1ml/hr flow rate. (F) Pareto Chart of the Effects for analysis of the factorial design shows that flow rate effects fiber diameter the most, although not significantly at the ranges tested.

The fabrication of tissue-engineered scaffolds that mimic the native 3D architecture of the ECM is necessary to facilitate the complex interactions that occur between cells and their microenvironment in native tissues. The process of electrospinning is a well-studied methodology for creating nanofiber scaffolds with desirable features, such as high surface area and porosity [5,24,25]. In this first experiment, we designed a two-level fractional factorial design of experiment (DOE) to determine the influence of the electrospinning process parameters of collecting distance, applied voltage, and flow rate on PNIPAM nanofiber morphology and diameter. The SEM images presented in Fig 1(A)–1(D) show the results of varying collecting distance and voltage at a flow rate of 0.1 ml/hr. As shown, nanofiber morphology is qualitatively comparable for the parameters tested, and the parameters yield uniform fibers in a random orientation. Fiber diameter was quantified for all groups and is presented in Fig 1E. There was no statistical difference in fiber diameter across the four different groups at 0.1 ml/hr (ANOVA). At 1 ml/hr flow rate, nanofibers electrospun at 15 kV, and 10 cm, were statistically greater than the other groups at that same flow rate. Analysis of the factorial design in Minitab yielded the Pareto chart of the effects, as shown in Fig 1F. A Pareto chart of the effects is useful for determining the magnitude and importance of an effect. Any effect that extends past the statistical reference line (α = 0.05) on the chart is considered significant [26]. As shown in Fig 1F, flow rate, voltage, or collecting distance do not significantly influence fiber diameter at the values tested. While not statistically significant, flow rate is shown to have the most impact on average fiber diameter. As a result, we elected to use the 0.1 ml/hr flow rate at 15 kV and 10cm for all studies moving forward, as the fiber morphology was uniform and average fiber diameter was in acceptable ranges–approximately 400-600nm.

**Fig 2:**
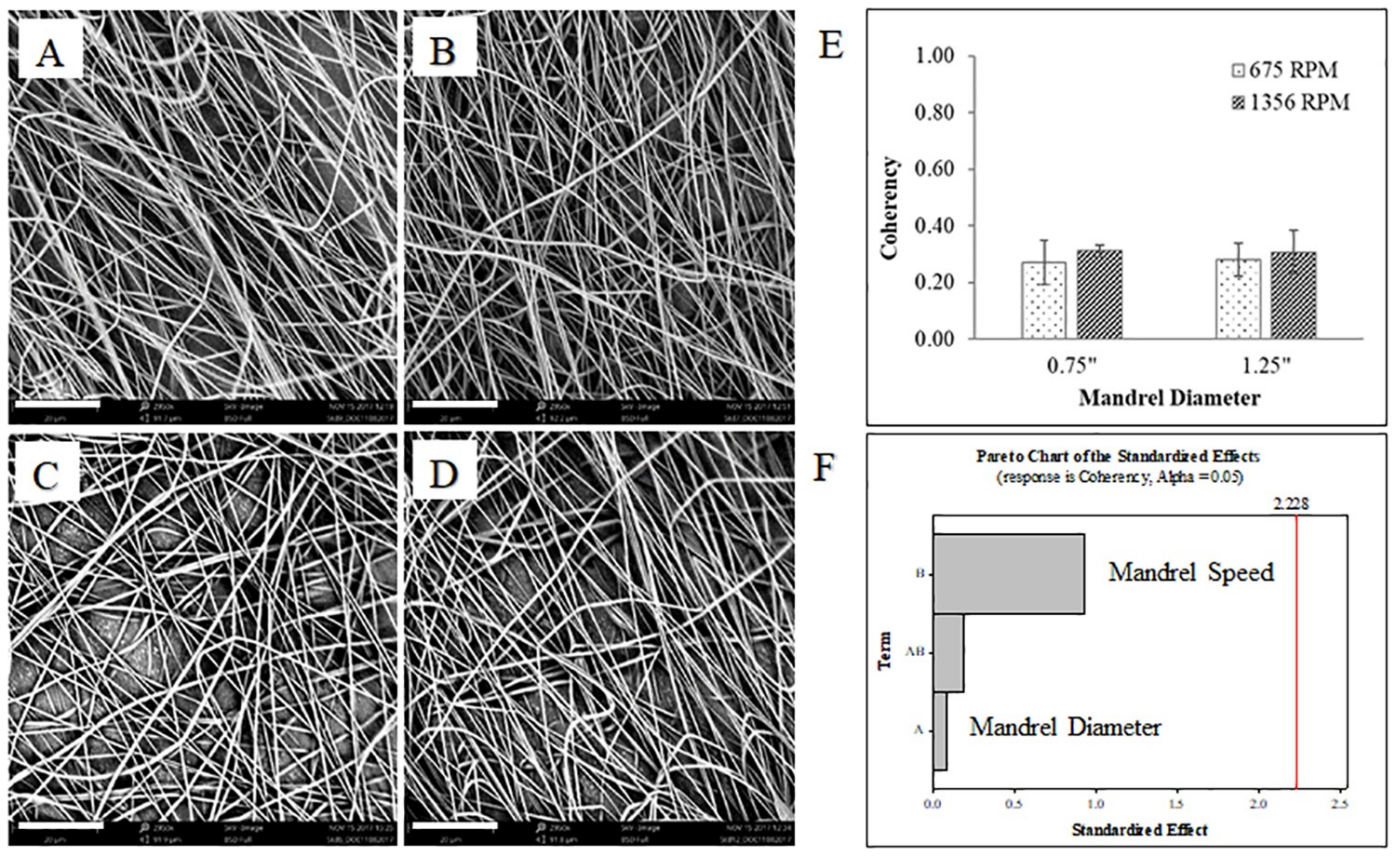
PNIPAM nanofibers as a function of mandrel diameter and mandrel speed. (A) 0.75” (19mm) mandrel diameter, 675 RPM. (B) 0.75”, 1356 RPM. (C) 1.25” (31.8mm), 675 RPM. (D) 1.25”, 1356 RPM. Scale bar (lower left of each image) is 20 μm. Alignment is not affected by mandrel diameter or mandrel speed, as shown in (E) as coherency values and (F) in the analysis of the factorial design.

Aligned nanofibers are advantageous for replicating the ECM in anisotropic tissues. This macro-level anisotropy of the uniaxially aligned fibers can provide guidance for cell growth, proliferation, migration, and differentiation [27,28]. Several techniques for aligning nanofibers have been reported in the literature, including electrospinning onto grounded parallel electrodes [24,29,30], rotating disk [31,32], and rotating drum [24,33,34]. In this study, we employed a rotating mandrel set-up and designed a two-level full factorial design of experiment (DOE) to determine if mandrel diameter or mandrel speed affect fiber orientation. The flow rate, voltage and collecting distance were held constant at 0.1 mL/hr, 15 kV, and 13 cm, respectively. The lower and upper limits of mandrel diameter and speed were 0.75 inches and 1.25 inches and 675 RPM and 1,356 RPM, respectively. The representative SEM images in Fig 2(A)–2(D) show uniform morphology, but no uniaxial alignment of nanofibers. Fiber alignment is quantified as a coherency value from Orientation J in Fig 2E; a coherency coefficient close to 1 indicates a strongly coherent orientation of fibers in the direction of the long axis. All coherency values are low—under 0.40 —indicating little alignment of the nanofibers in the scaffold. The Pareto chart in Fig 2F shows no statistical significance of the mandrel diameter or speed on coherency factor, as tested at an alpha level of 0.05. However, the Pareto chart does indicate that mandrel speed may have a greater influence on coherency factor, as shown by the factor bar having the highest standardized effect.

**Fig 3:**
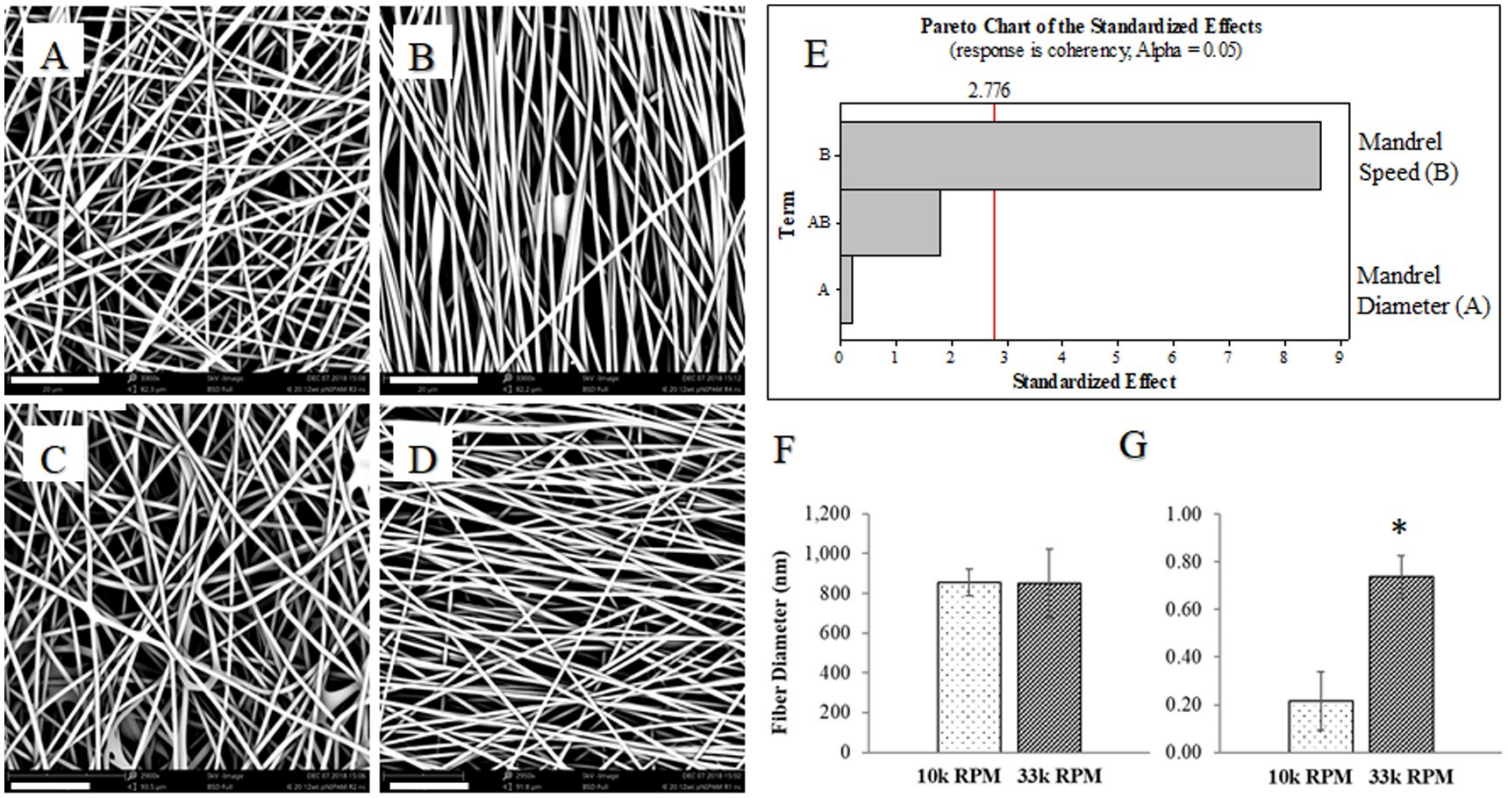
Effect of mandrel diameter and speed on fiber diameter and coherency. (A) 0.156” (4mm) mandrel diameter, 10,000 RPM. (B) 0.156”, 33,000 RPM. (C) 0.195”, 10,000 RPM. (D) 0.195”, 33,000 RPM. (E) Factorial design analysis shows mandrel speed to be a significant factor on Coherency (alignment). (F) Fiber diameter and (G) Coherency of nanofibers spun at 33,000 RPM and mandrel diameter 0.156”. Scale bar (lower left of each image) is 20 μm. *p<0.05 by 2-sample t-test.

To test the hypothesis generated from the results in Fig 2, we used another two-level factorial DOE to determine the effect of greater mandrel speeds on nanofiber alignment. A Dremel 300 was used for its range of speeds: 10-33k RPM. However, the Dremel chuck is more limited in size, so only a very narrow range of mandrel diameters could be tested safely. The lower and upper limits of mandrel diameter and speed were 0.156 inches and 0.195 inches and 10,000 RPM and 33,000 RPM, respectively. Representative SEM micrographs in Fig 3(A)–3(D) qualitatively demonstrate that alignment is achievable at the highest mandrel speed (Fig 2B and 2D). The Pareto chart in Fig 3E confirms the statistical significance of mandrel speed on nanofiber alignment, represented by the coherency factor from Orientation J. Average fiber diameter for the PNIPAM scaffold electrospun on the 0.156 inch diameter mandrel is shown in Fig 3F. Importantly, fiber diameters of scaffolds electrospun at mandrel speeds of 10,000 and 33,000 RPM were found to be statistically similar; this will prove important in future studies when comparing the cellular effects on random vs. aligned fibers. Alignment of the PNIPAM scaffold electrospun on the 0.156 inch diameter mandrel is presented in Fig 3G as the coherency factor from Orientation J. Scaffolds electrospun at a mandrel speed of 33,000 RPM had a significantly higher coherency factor (0.74)–indicating a high degree of alignment for a scaffold electrospun on a high speed mandrel. These results indicate that alignment of PNIPAM nanofibers is dependent on the speed of the collecting mandrel. A high degree of nanofiber alignment obtainable at high collecting mandrel speeds, while still maintaining a desirable fiber diameter.

PNIPAM has been studied extensively for various biomedical applications ranging from drug delivery to tissue engineering. The polymer is typically fabricated into a thermoresponsive gel, and only recently has the idea of electrospinning PNIPAM come into the literature. Several groups have examined the effect of polymer solution concentration, composition and solvent choice on PNIPAM fiber diameter [9,12,35]. All of the groups reported a “dog bone” morphology in the cross sections of the electrospun PNIPAM fibers, attributing this phenomenon to the electric charge distribution in fibers during electrospinning [9]. Molecular weight of the polymer reflects the entanglement of polymer chains in solution, or the solution viscosity, and has been shown to have an important effect on fiber morphology [25,36]. All groups report the feasibility of electrospinning nonwoven scaffolds of HMW PNIPAM, composed of fibers with average diameters on the order of microns [9,12,35]. Alignment of nanofibers is desirable to drive a certain cell phenotype and encourage growth of cells in the direction of alignment. Previously, Edwards et al. examined the influence of a rotating collector on the internal and external structure of electrospun PCL fibers, reporting reduced fiber diameter as speed of the collector increases and the ability to control fiber structure for specific applications [37]. More recently, Allen *et al*. reported on aligning PNIPAM/PCL polymer blends by electrospinning onto a rotating mandrel, achieving fiber alignment at varying concentrations of PCL; however their studies only included a narrow range of rotating speeds, and their fibers maintained diameters in the micron range a [12]. Here we report electrospun PNIPAM fibers (using the Dremel) with a uniform morphology and diameters in the range of 700–900 nm, which more closely mimics native ECM components with the goal to enhance cell adhesion [38]. We provide a method for aligning PNIPAM nanofibers without chemical modification or use of another polymer. The results further provide an understanding of the effect of mandrel diameter and speed on the alignment of nanofibers in the scaffold.

### Nanofiber crosslinking and characterization

**Fig 4:**
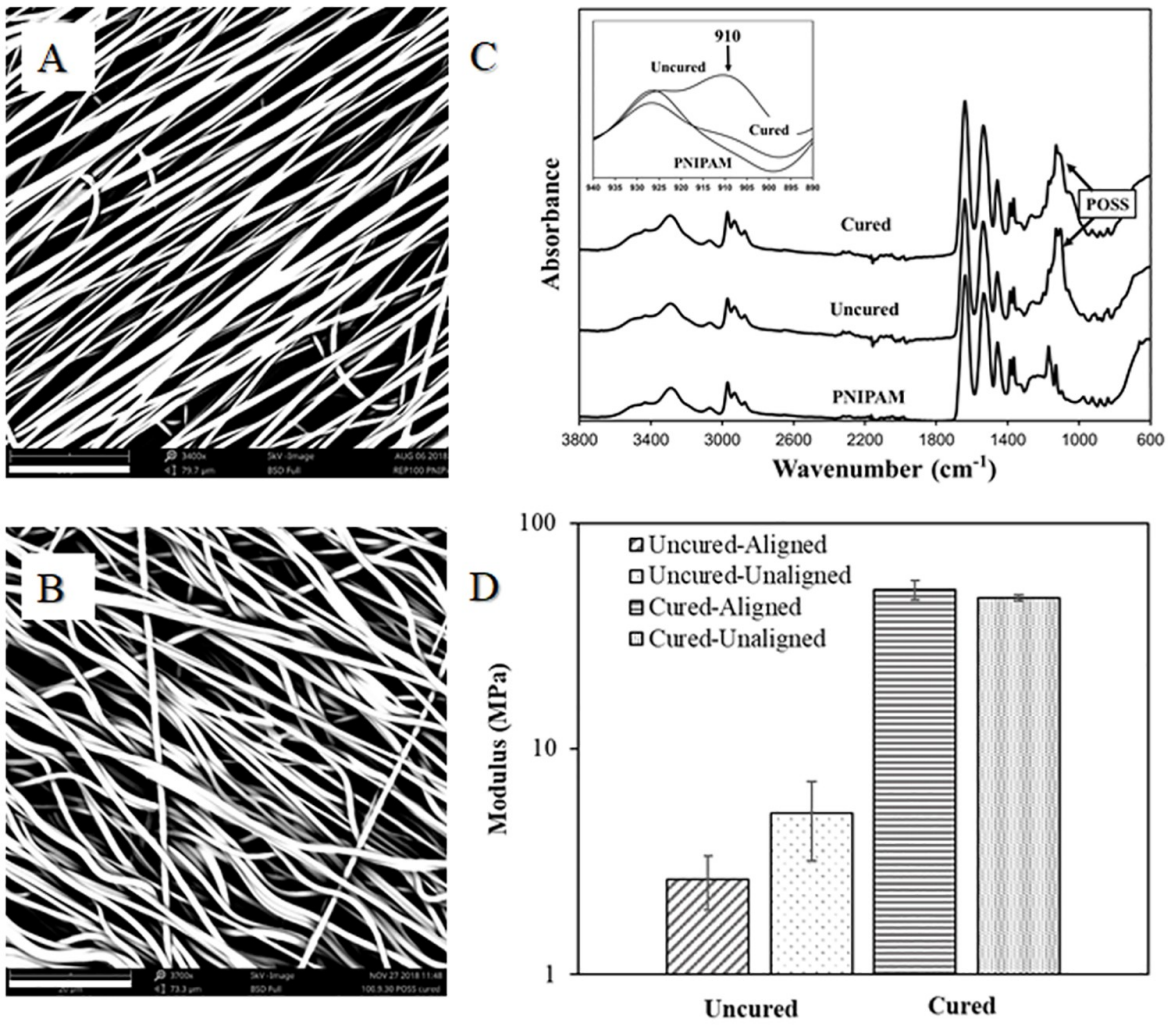
PNIPAM nanofiber morphology before and after curing. (A) Aligned nanofibers before heat treatment. (B) Aligned nanofibers after heat treatment. Scale bar (lower left of each SEM image) is 20 μm. (C) FTIR spectra of PNIPAM-only fibers and PNIPAM:OpePOSS:EMI fibers before and after curing. Characteristic OpePOSS peaks at 1110 cm^-1^ are present in the cured and uncured samples. After curing, the epoxide peak at 910 cm^-1^ disappears (inset). (D) Modulus of aligned and unaligned nanofiber scaffolds increases significantly after curing.

PNIPAM has shown to be soluble in aqueous solutions [1]; therefore, to maintain stability in cell culture, we chemically crosslinked the electrospun PNIPAM scaffolds. SEM micrographs in Fig 4A and 4B depict the nanofiber morphology before and after curing at 120°C for 4 hours. Fibers maintain uniformity, but develop a characteristic waviness. ATR-FTIR spectroscopy was used to confirm the crosslinking reaction (Fig 4C). A PNIPAM nanofiber scaffold electrospun without OpePOSS and EMI in solution was used as a negative control. As seen in the spectra, the PNIPAM sample lacked the characteristic OpePOSS peak at 1110 cm^-1^, which was present in both the uncured and cured samples. Additionally, as shown in Fig 4C inset, the characteristic epoxide peak at 910 cm^-1^ disappears after heat treatment, confirming the crosslinking via ring-opening of epoxide groups in OpePOSS [39]. To verify if the crosslinking reaction occurred beyond the surface of the scaffold, FTIR spectra of PNIPAM:OpePOSS:EMI scaffolds before and after curing were obtained in transmission mode. Similar to the ATR-FTIR spectra, the epoxide peak at 910 cm^-1^ disappeared after curing, indicating that the crosslinking reaction occurred throughout the scaffold (S1 Fig) Based on this measurement and the 100:20 PNIPAM:OpePOSS ratio used in this study, the percent crosslinking of the -NH groups on the PNIPAM is approximately 14%, which is slightly higher than that reported by Wang et al., an expected result given the 100:15 PNIPAM:OpePOSS ratio used in their study. Nearly identical FTIR spectra were acquired in various locations in the same cured sample, indicating a high level of spatial uniformity in our samples.

The mechanical properties of the scaffolds, namely storage modulus (MPa), were quantified using dynamic mechanical analysis (DMA). As shown in Fig 4D, the modulus increased significantly (by an order of magnitude) after curing. There was no significant difference between aligned and unaligned samples in either uncured scaffolds or cured scaffolds. Due to limitations in the size of scaffolds produced on the Dremel 300 mandrel (4mm diameter), the modulus was not measured in the uniaxial direction of alignment. Although not statistically significant (p = 0.087), the unaligned uncured sample had a higher modulus than the aligned uncured scaffold, confirming the difference in fiber morphology (physical entanglement of nanofibers). In aligned nanofiber scaffolds, the modulus increased significantly after curing (measured perpendicular to alignment direction), confirming crosslinking between adjacent fibers [40,41].

**Fig 5:**
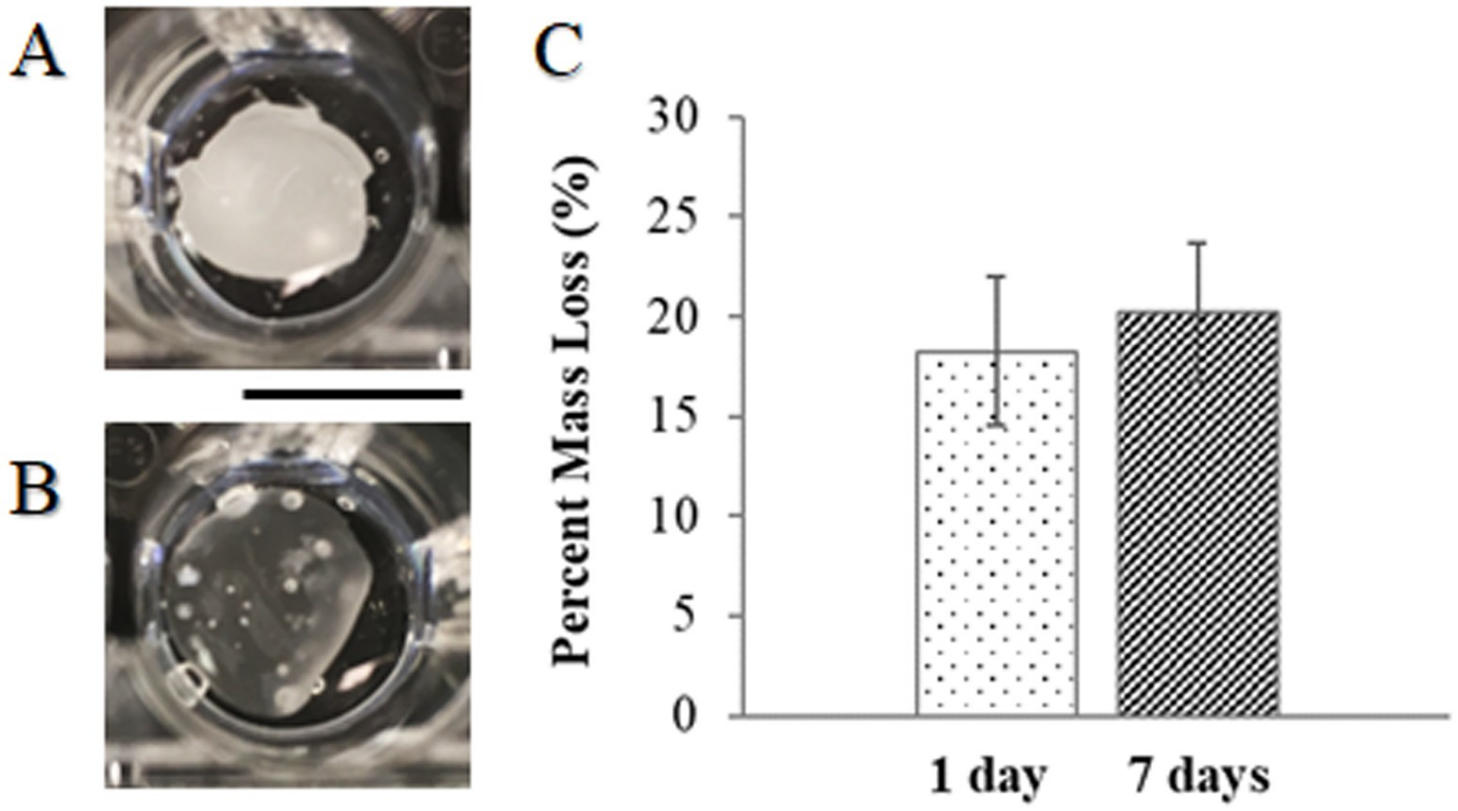
Aqueous stability and observable phase transition of cured PNIPAM nanofiber scaffolds after 1 or 7 days of immersion in deionized water. Qualitative scaffold images taken at 24 hours at 37°C above LCST (A) and 22°C, below LCST (B). Scale bar is 1cm. Aqueous stability is quantified as percent mass loss (C). Mass loss does not change significantly with increasing time in culture (p = 0.710).

To confirm crosslinking and determine aqueous stability over time, the cured PNIPAM scaffolds were immersed in deionized water and incubated overnight. Fig 5 shows the scaffolds immediately after being removed from the incubator at 37°C (A), and after cooling to 22°C (B), which took about 15 minutes. The scaffolds were opaque at 37°C, because of being above the polymer LCST in a collapsed, hydrophobic state. The scaffolds maintained their physical shape (i.e. minimal contraction) after cooling and appeared relatively translucent, because of being below the polymer LCST in a swelled, hydrophilic state. Notably, these nanofiber scaffolds were cycled through thermoresponsive phase changes once a day for 7 consecutive days to determine long-term stability for cell culture. The scaffolds continually cycled through the phase transitions with no observable changes in scaffold size or shape, demonstrating feasibility of use for tissue engineering applications. Mass loss of the scaffolds was measured after 1 and 7 days of immersion in deionized water. The average percent mass loss was not statistically different between 1 and 7 days in culture, as shown in Fig 5C. Since PNIPAM is not biodegradable [42], the observed percent mass loss may be attributed to fraying edges of nanofibers after the scaffolds were cut for experimentation.

PNIPAM has been electrospun previously in combination with other biocompatible and biodegradable polymers for use in cell culture applications [11,12]. However, pure electrospun PNIPAM has had limited success [10] for *in vitro* applications because it solubilizes in aqueous solutions due to its imperfect hydrophobicity [43]. Cicoette *et al*. reported the presence of thermoresponsive properties in HMW PNIPAM scaffolds electrospun in methanol [10]. Swelling and collapsing of the polymer scaffold, below and above the LCST, was observed with an inverted optical microscope to show the thermoresponsive properties of the scaffold and the stability in aqueous solutions [10]. Maeda et al. also electrospun HMW PNIPAM fiber meshes and demonstrated dynamic shrinking behavior upon heating above the LCST due to the high surface area and porosity of the meshes. Although very promising as a temperature-modulated cell capture and release system, the dynamic shrinking of the scaffold is problematic for cell sheet growth [36]. Slemming-Adamsen *et al*. developed a method using N-hydroxysuccinimide (NHS) and N-(3-dimethylaminopropyl)-N’ethylcarbodiimide hydrochloride (EDC) to chemically crosslink an electrospun scaffold made of gelatin and an N-hydroxysuccinimide ester terminated PNIPAM chain [44]. The scaffolds exhibited thermoresponsive properties by undergoing conformational changes above and below the LCST; however the fiber size changed, shrinking from 700 nm to 400 nm when heated to 37°C. Furthermore, NHS ester-terminated PNIPAM is not commercially available without custom synthesis, while the crosslinking methodology using OpePOSS is simple to incorporate, inexpensive, and readily available. Allen *et al*. observed greater than 75% mass loss for scaffolds containing 90% or more PNIPAM, which is not surprising without chemical crosslinking [12]. To this end, the present study expands upon the crosslinking methodology reported by Wang et al. to align PNIPAM nanofibers in the nanometer range without the use of additional polymers [14].

### Cell culture applications

**Fig 6:**
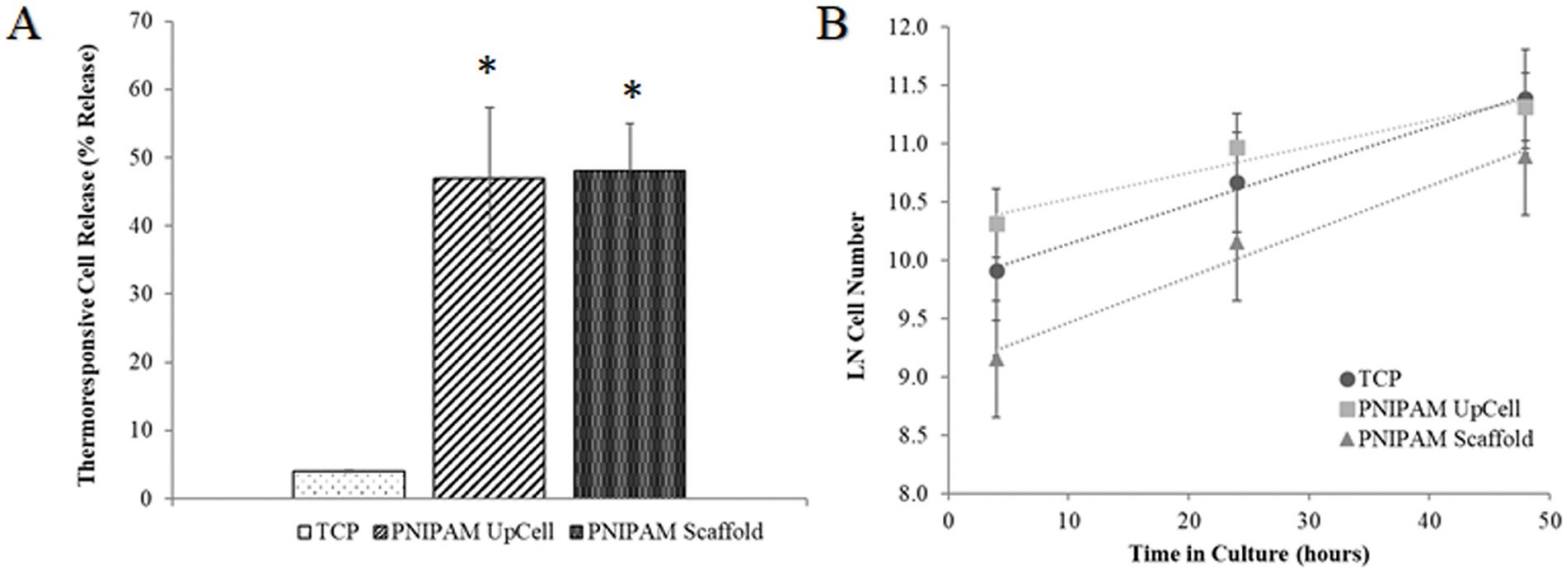
L929 cell attachment, thermorelease, and proliferation on cured PNIPAM nanofiber scaffolds compared to TCP and Nunc PNIPAM UpCell Surface. (A) Thermoresponsive release of L929 cells after 16 hours in culture. Cured PNIPAM nanofiber scaffolds were statistically similar compared to commercially available Nunc PNIPAM UpCell Surface. Tissue culture plastic (TCP) was used as a negative control. (*p < 0.05 compared to TCP). (B) L929 cell attachment and proliferation on cured PNIPAM nanofiber scaffolds compared to TCP and Nunc PNIPAM UpCell Surface. Cell growth rates (a comparison of slopes) were statistically similar on all substrates. (p = 0.169 by General Linear Model Regression).

To further examine the thermoresponsive properties of the scaffold, the release of L-929 fibroblasts was measured after 16 hours of initial attachment to the electrospun PNIPAM scaffold, UpCell, and TCP surfaces. Fig 6A quantifies the percentage of cellular release as a function of culture surface. The UpCell surface and electrospun PNIPAM scaffold exhibited no significant difference in thermoresponsive cellular release; however, as expected, both thermoresponsive substrates released statistically more cells than the TCP negative control.

UpCell temperature-responsive cell culture dishes have become the standard of practice in the field of cell sheet engineering. From a technical bulletin produced by the company, the acceptable detachment from UpCell is 50% [45]. Due to their comparable thermoresponsive cell release, electrospun PNIPAM scaffolds could become a reasonable alternative to UpCell for cell sheet harvesting. Additionally, electrospun PNIPAM scaffolds offer the advantage of a 3D architecture more closely replicating ECM and the potential for anisotropic tissue formation.

Following confirmation that the scaffolds could support initial cell attachment and release cells in response to a temperature-induced phase shift, we cultured L-929 cells on the scaffolds over 48 hours to determine longer-range biocompatibility and quantify cell proliferation. As described above, TCP and UpCell were used as control groups. In Fig 6B, cell density of L-929 cells was measured at 4, 24, and 48 hours of culture on PNIPAM scaffolds, UpCell, and TCP and plotted as a natural log of cell number. Statistical analysis shows that cell density is statistically different between all three surfaces at 4 and 24 hours. At 48 hours, cell density converges, yet the cell density on PNIPAM remains significantly lower. Because our nanofiber scaffolds contain OpePOSS, it is important to note that OpePOSS has been confirmed to be non-cytotoxic and biocompatible [46]. POSS was synthesized to improve the thermal response rates of PNIPAM hydrogels and has been shown to enhance glass transition temperatures, as well as thermal stability when compared to plain PNIPAM networks [47]. A recent study by Tong et al. also demonstrated enhanced cell adhesion and proliferation on POSS-PNIPAM hydrogel scaffolds compared to PNIPAM hydrogels without POSS [48].

It is reasonable to interpret the 4-hour time-point as the initial level of attachment. Due to lower initial cell density on PNIPAM scaffolds, we used the linearized form of the growth equation to examine cell growth over time, as shown in the following equation:

$$\text{ln}\;\left(X\right)=\text{ln}\;\left(X_{0}\right)+\mu t,$$

where X is the concentration of the cells on the substrate at time t, X_0_ is the initial concentration of cells adhered to the substrate, and μ is the cell growth rate.

Fig 6B plots the linearized growth equation to more easily compare the growth rates, as shown by the slopes of the linear trendlines. Using regression analysis, the slopes were determined to be 0.039, 0.0334, and 0.0224 for the PNIPAM Scaffold, UpCell, and TCP, respectively. The General Linear Model Regression Analysis in Mintiab was used to compare these slopes. The p-value for the general regression analysis was 0.169, indicating the growth rate of cells on each scaffold is not statistically different. To this end, data presented in Fig 6 confirm that OpePOSS-containing PNIPAM nanofiber scaffolds are thermoresponsive and capable of supporting fibroblast cell growth over time, albeit with a lower initial cell density. To circumvent this low initial attachment rate and improve cell adhesion, PNIPAM nanofibers could be coated with ECM proteins, such as Matrigel [12], fibronectin [28], or collagen [49].

### Conclusions

In this study, we employ factorial design of experiments to systematically determine the effects of different electrospinning process parameters on PNIPAM nanofiber diameter and alignment. Here, we present the range of parameters tested and aim to highlight the robustness of a statistical DOE approach in identifying an optimal parameter set. We demonstrate that HMW PNIPAM can be electrospun into random or uniaxially aligned nanofiber mats by simply altering speed of the rotating mandrel collector. Importantly, fiber diameter was the same for random vs. aligned fibers, allowing for an accurate comparison in future studies that examine the effects of alignment on cell function and phenotype. The optimized cross-linked nanofibers were stable in aqueous culture, biocompatible, and thermoresponsive. The PNIPAM scaffolds fabricated in the present study show promise for tissue engineering applications where uniaxial alignment is ideal, such as vascular, bone, or neural grafts.

## Supporting information

S1 Fig FTIR spectra PNIPAM:OpePOSS:EMI fibers before and after curing. After curing, the epoxide peak at 910 cm^-1^ disappears, indicating that the crosslinking reaction occurred throughout the fiber scaffold.
(TIF)

## References

[1] Schild HG. Poly(N-isopropylacrylamide): experiment, theory and application. Prog Polym Sci. 1992; 17(2): 163–249.

[2] Canavan H, Cheng X, Graham D, Ratner B, Castner D. Cell sheet detachment affects the extracellular matrix: a surface science study comparing thermal liftoff, enzymatic, and mechanical methods. J Biomed Mater Res A. 2005; 75(1): 1–13. 10.1002/jbm.a.30297 16086418

[3] Kushida A, Yamato M, Kikuchi A, Sakurai Y, Okano T. Decrease in culture temperature releases monolayer endothelial cell sheets together with deposited fibronectin matrix from temperature-responsive culture surfaces. J Biomed Mater Res. 1999; 45(4): 355–62. 10321708 10.1002/(sici)1097-4636(19990615)45:4<355::aid-jbm10>3.0.co;2-7

[4] Canavan H, Cheng X, Graham D, Ratner B, Castner D. Surface characterization of the extracellular matrix remaining after cell detachment from a thermoresponsive polymer. Langmuir. 2004; 21(5): 1949–55. 10.1021/la048546c 15723494

[5] Liao S, Li B, Ma Z, Wei H, Chan C, Ramakrishna S. Biomimetic electrospun nanofibers for tissue regeneration. Biomed Mater. 2006; 1(3): R45 10.1088/1748-6041/1/3/R01 18458387

[6] Martins-Green M. The dynamics of cell–ECM interactions with implications for tissue engineering In: Lanza R, Langer R, Chick W, editors. Principles of Tissue Engineering. 4th ed San Diego, CA: Academic Press; 1997 p. 23–46.

[7] Stevens MM, George JH. Exploring and engineering the cell surface interface. Science. 2005; 310(5751): 1135–8. 10.1126/science.1106587 16293749

[8] Tang Z, Akiyama Y, Okano T. Temperature-Responsive Polymer Modified Surface for Cell Sheet Engineering. Polymers. 2012; 4(3): 1478–98.

[9] Rockwood D, Chase D, Akins R Jr., Rabolt J. Characterization of electrospun poly(N-isopropyl acrylamide) fibers. Polymer. 2008; 49(18): 4025–32.

[10] Cicotte K, Reed J, Nguyen P, De Lora J, Hedberg-Dirk E, Canavan H. Optimization of electrospun poly(N-isopropyl acrylamide) mats for the rapid reversible adhesion of mammalian cells. Biointerphases. 2017; 12(2). 10.1116/1.4984933 PMC5469682 28610429

[11] Kim M, Lee H, Bang S, Yang H, Kang S, Suh K, et al 3D tissue formation by stacking detachable cell sheets formed on nanofiber mesh. Biofabrication. 2017; 9(1). 10.1088/1758-5090/aa64a0 28332479

[12] Allen A, Barone E, Crosby C, Suggs L, Zoldan J. Electrospun poly(N-isopropyl acrlyamide)/poly(caprolactone) fibers for the generation of anisotropic cell sheets. Biomater Sci. 2017; 5(8): 1661–9. 10.1039/c7bm00324b 28675203 PMC5870125

[13] Nakayama M, Okano T, Winnik F. Poly(N-isopropylacrylamide)-based Smart Surfaces for Cell Sheet Tissue Engineering. Material Matters. 2010; 5.3(56).

[14] Wang J, Sutti A, Wang X, Lin T. Fast responsive and morphologically robust thermo-responsive hydrogel nanofibres from poly(N-isopropylacrylamide) and POSS crosslinker. Soft Matter. 2011; 7(9): 4364–9.

[15] Sensini A, Cristofolini L. Biofabrication of Electrospun Scaffolds for the Regeneration of Tendons and Ligaments. Materials. 2018; 11(10): 1963. 10.3390/ma11101963 PMC6213815 30322082

[16] Lee CH, Shin HJ, Cho IH, Kang Y-M, Kim IA, Park K-D, et al Nanofiber alignment and direction of mechanical strain affect the ECM production of human ACL fibroblast. Biomaterials. 2005; 26(11): 1261–70. 10.1016/j.biomaterials.2004.04.037 15475056

[17] Schnell E, Klinkhammer K, Balzer S, Brook G, Klee D, Dalton P, et al Guidance of glial cell migration and axonal growth on electrospun nanofibers of poly-ε-caprolactone and a collagen/poly-ε-caprolactone blend. Biomaterials. 2007; 28(19): 3012–25. 10.1016/j.biomaterials.2007.03.009 17408736

[18] Seyedmahmoud R, Rainer A, Mozetic P, Maria Giannitelli S, Trombetta M, Traversa E, et al A primer of statistical methods for correlating parameters and properties of electrospun poly (l -lactide) scaffolds for tissue engineering-PART 1: Design of experiments: Methods for Correlating Parameters and Properties of Electrospun Scaffolds. J Biomed Mater Res A. 2015; 103(1): 91–102. 24616399 10.1002/jbm.a.35153

[19] Ruiter FAA, Alexander C, Rose FRAJ, Segal JI. A design of experiments approach to identify the influencing parameters that determine poly-D,L-lactic acid (PDLLA) electrospun scaffold morphologies. Biomed Mater. 2017; 12(5): 055009 10.1088/1748-605X/aa7b54 28643700

[20] Coles SR, Jacobs DK, Meredith JO, Barker G, Clark AJ, Kirwan K, et al A design of experiments (DoE) approach to material properties optimization of electrospun nanofibers. J Appl Polym Sci. 2010; 117(4): 2251–7.

[21] Costolo MA, Lennhoff JD, Pawle R, Rietman EA, Stevens AE. A nonlinear system model for electrospinning sub-100 nm polyacrylonitrile fibres. Nanotechnology. 2008; 19(3): 035707 10.1088/0957-4484/19/03/035707 21817592

[22] Fonck E, Feigl GG, Fasel J, Sage D, Unser M, Rüfenacht DA, et al Effect of aging on elastin functionality in human cerebral arteries. Stroke. 2009; 40(7): 2552–6. 10.1161/STROKEAHA.108.528091 19478233

[23] Sefcik LS, Kaminski A, Ling K, Laschewsky A, Lutz J-F, Wischerhoff E. Effects of PEG-Based Thermoresponsive Polymer Brushes on Fibroblast Spreading and Gene Expression. Cell Mol Bioeng. 2013; 6(3): 287–98.

[24] Teo W, Kotaki M, Mo X, Ramakrishna S. Porous tubular structures with controlled fibre orientation using a modified electrospinning method. Nanotechnology. 2005; 16:918–24.

[25] Li Z, Wang C. Effects of Working Parameters on Electrospinning In: One-Dimensional Nanostructures. 2013 p. 15–28.

[26] Getting Started with Minitab 18 page 47. https://www.minitab.com/uploadedFiles/Documents/getting-started/Minitab18-GettingStarted-EN.pdf

[27] Mitchell GR, Tojeira A. Role of Anisotropy in Tissue Engineering. Procedia Eng. 2013; 59: 117–25.

[28] Takahashi H, Okano T. Cell Sheet-Based Tissue Engineering for Organizing Anisotropic Tissue Constructs Produced Using Microfabricated Thermoresponsive Substrates. Adv Healthc Mater. 2015; 4(16): 2388–407. 10.1002/adhm.201500194 26033874

[29] Li D, Wang Y, Xia Y. Electrospinning of Polymeric and Ceramic Nanofibers as Uniaxially Aligned Arrays. Nano Lett. 2003; 3(8): 1167–71.

[30] Park SH, Yang D-Y. Fabrication of aligned electrospun nanofibers by inclined gap method. J Appl Polym Sci. 2011; 120(3): 1800–7.

[31] Ghasemi-Mobarakeh L, Prabhakaran MP, Morshed M, Nasr-Esfahani M-H, Ramakrishna S. Electrospun poly(ɛ-caprolactone)/gelatin nanofibrous scaffolds for nerve tissue engineering. Biomaterials. 2008; 29(34): 4532–9. 10.1016/j.biomaterials.2008.08.007 18757094

[32] Xu C. Aligned biodegradable nanofibrous structure: a potential scaffold for blood vessel engineering. Biomaterials. 2004; 25(5): 877–86. 14609676 10.1016/s0142-9612(03)00593-3

[33] Lee CH, Shin HJ, Cho IH, Kang Y-M, Kim IA, Park K-D, et al Nanofiber alignment and direction of mechanical strain affect the ECM production of human ACL fibroblast. Biomaterials. 2005; 26(11): 1261–70. 10.1016/j.biomaterials.2004.04.037 15475056

[34] Meng Z, Wang Y, Ma C, Zheng W, Li l, Zheng Y. Electrospinning of PLGA/gelatin randomly-oriented and aligned nanofibers as potential scaffold in tissue engineering. Mater Sci Eng. 2010; 30(8): 1204–10.

[35] Okuzaki H, Kobayashi K, Yan H. Non-woven fabric of poly(N-isopropylacrylamide) nanofibers fabricated by electrospinning. Synth Met. 2009; 159(21–22): 2273–6.

[36] Maeda T, Kim Y-J, Aoyagi T, Ebara M. The Design of Temperature-Responsive Nanofiber Meshes for Cell Storage Applications. Fibers. 2017; 5(1): 13.

[37] Edwards MD, Mitchell GR, Mohan SD, Olley RH. Development of orientation during electrospinning of fibres of poly(ε-caprolactone). Eur Polym J. 2010; 46(6): 1175–83.

[38] Rhodes JM, Simons M. The extracellular matrix and blood vessel formation: not just a scaffold. J Cell Mol Med. 2007; 11(2): 176–205. 10.1111/j.1582-4934.2007.00031.x 17488472 PMC3822822

[39] Wang J, Sutti A, Lin T, Wang X. Thermo-responsive PNIPAM hydrogel nanofibres photocrosslinked by azido-POSS. Proc SPIE Int Soc Opt Eng. 2013;

[40] Tan AR, Ifkovits JL, Baker BM, Brey DM, Mauck RL, Burdick JA. Electrospinning of photocrosslinked and degradable fibrous scaffolds. J Biomed Mater Res A. 2008; 87A(4): 1034–43. 10.1002/jbm.a.31853 PMC5561734 18257065

[41] Jose M, Thomas V, Johnson K, Dean D, Nyairo E. Aligned PLGA/HA nanofibrous nanocomposite scaffolds for bone tissue engineering. Acta Biomater. 2009; 5(1): 305–15. 10.1016/j.actbio.2008.07.019 18778977

[42] Cui Z, Lee BH, Vernon BL. New Hydrolysis-Dependent Thermosensitive Polymer for an Injectable Degradable System. Biomacromolecules. 2007; 8(4): 1280–6. 10.1021/bm061045g 17371066 PMC2892927

[43] Pelton R. Poly(N-isopropylacrylamide) (PNIPAM) is never hydrophobic. J Colloid Interface Sci. 2010; 348(2): 673–4. 10.1016/j.jcis.2010.05.034 20605160

[44] Slemming-Adamsen P, Song J, Dong M, Besenbacher F. In Situ Cross-Linked PNIPAM/Gelatin Nanofibers for Thermo-Responsive Drug Release. Macromol Mater Eng. 2015; 12: 1226–31.

[45] Thermofisher. Cell Harvesting by Temperature Reduction. Technical Bulletin page12. https://www.thermofisher.com/content/dam/LifeTech/latin-america/promotions/images/2016/Q4/Cell%20Harvesting%20by%20Temperature%20Reduction_Thermo%20Scientific%20Nunc%20UpCell%20Surface.pdf

[46] Shen C, Han Y, Wang B, Tang J, Chen H, Lin Q. Ocular biocompatibility evaluation of POSS nanomaterials for biomedical material applications. RSC Adv. 2015; 5(66): 53782–8. 10.1039/d4ra90030h PMC10975251 38549796

[47] Zeng K, Fang Y, Zheng S. Organic–Inorganic Hybrid Hydrogels Involving Poly(N-isopropylacrylamide) and Polyhedral Oligomeric Silsesquioxane: Preparation and Rapid Thermoresponsive Properties. J Polym Sci B Polym Phys. 2009; 47(5): 504–16.

[48] Tong Y, Zhang Y, Liu Y, Cai H, Zhang W, Tan W-S. POSS-enhanced thermosensitive hybrid hydrogels for cell adhesion and detachment. RSC Adv. 2018; 8(25): 13813–9. 10.1039/c8ra01584h PMC9079822 35539329

[49] Moran MT, Carroll WM, Selezneva I, Gorelov A, Rochev Y. Cell growth and detachment from protein-coated PNIPAAm-based copolymers. J Biomed Mater Res A. 2007; 81A(4): 870–6. 10.1002/jbm.a.31089 17236213

