# Supplementary figures and images for: Optimizing the alignment of thermoresponsive poly(N-isopropyl acrylamide) electrospun nanofibers for tissue engineering applications: A factorial design of experiments approach
Source: PLoS One. 2019 Jul 5;14(7):e0219254. doi: 10.1371/journal.pone.0219254 (PMC6611625; doi:10.1371/journal.pone.0219254)

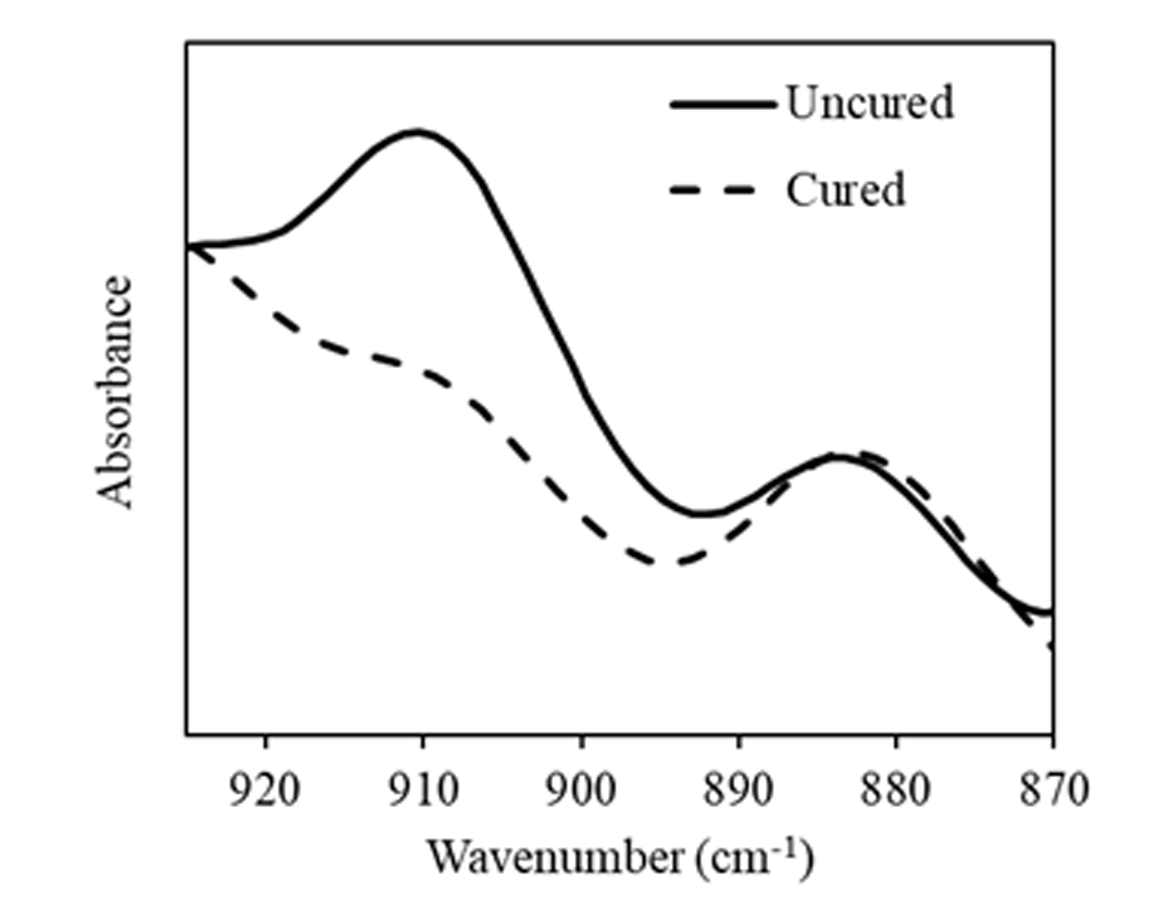

Supplement: S1 Fig — After curing, the epoxide peak at 910 cm-1 disappears, indicating that the crosslinking reaction occurred throughout the fiber scaffold. (TIF) [file pone.0219254.s001.tif]
